# Functional divergence of the bitter receptor TAS2R38 in Sulawesi macaques

**DOI:** 10.1002/ece3.5557

**Published:** 2019-08-20

**Authors:** Kanthi Arum Widayati, Xiaochan Yan, Nami Suzuki‐Hashido, Akihiro Itoigawa, Laurentia Henrieta Permita Sari Purba, Fahri Fahri, Yohey Terai, Bambang Suryobroto, Hiroo Imai

**Affiliations:** ^1^ Department of Biology Bogor Agricultural University Bogor Indonesia; ^2^ Wildlife Research Center Kyoto University Kyoto Japan; ^3^ Primate Research Institute Kyoto University Inuyama Japan; ^4^ Academy of Emerging Science Chubu University Kasugai Japan; ^5^ Department of Biology Tadulako University Palu Indonesia; ^6^ Department of Evolutionary Studies of Biosystems The Graduate University for Advanced Studies Hayama Japan

**Keywords:** adaptation, allopatric species, bitter taste receptor, Sulawesi macaque, TAS2R38

## Abstract

**Abstract:**

Bitter perception is mediated by G protein‐coupled receptors TAS2Rs and plays an important role in avoiding the ingestion of toxins by inducing innate avoidance behavior in mammals. One of the best‐studied TAS2Rs is TAS2R38, which mediates the perception of the bitterness of synthetic phenylthiocarbamide (PTC). Previous studies of TAS2R38 have suggested that geographical separation enabled the independent divergence of bitter taste perception. The functional divergence of TAS2R38 in allopatric species has not been evaluated. We characterized the function of TAS2R38 in four allopatric species of Sulawesi macaques on Sulawesi Island. We found variation in PTC taste perception both within and across species. In most cases, TAS2R38 was sensitive to PTC, with functional divergence among species. We observed different truncated TAS2R38s that were not responsive to PTC in each species of *Macaca nigra* and *M. nigrescens* due to premature stop codons. Some variants of intact TAS2R38 with an amino acid substitution showed low sensitivity to PTC in *M. tonkeana*. Similarly, this intact TAS2R38 with PTC‐low sensitivity has also been found in humans. We detected a shared haplotype in all four Sulawesi macaques, which may be the ancestral haplotype of Sulawesi macaques. In addition to shared haplotypes among Sulawesi macaques, other TAS2R38 haplotypes were species‐specific. These results implied that the variation in TAS2R38 might be shaped by geographical patterns and local adaptation.

**OPEN RESEARCH BADGES:**

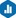

This article has earned an Open Data Badge for making publicly available the digitally‐shareable data necessary to reproduce the reported results. The data is available at https://doi.org/10.5061/dryad.908jf3r.

## Graphical abstract

We characterized the function of TAS2R38 in four allopatric species of Sulawesi macaques on Sulawesi Island. We found that TAS2R38 of Sulawesi macaques shows functional divergence with respect to phenylthiocarbamide responses, and the differences may reflect adaptation to local environmental conditions.

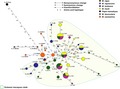

## 1 INTRODUCTION

Bitter taste perception is important for avoiding the ingestion of toxins by inducing innate avoidance behavior in mammals and is mediated by the G protein‐coupled receptors TAS2Rs, expressed in the membranes of sensory cells (Chandrashekar, Hoon, Ryba, & Zuker, 2006; Chandrashekar et al., 2000). TAS2Rs are also expressed in extra‐oral cells and may have roles in innate immunity (Lee et al., 2012; Shaw et al., 2018). One of the best‐studied TAS2Rs is TAS2R38, which mediates the perception of the bitterness of synthetic phenylthiocarbamide (PTC), propylthiouracil, and natural bitter compounds, such as allyl isothiocyanate, goitrin, limonin, yohimbine, and sinigrin (Meyerhof et al., 2010).

Correlations between the divergence of *TAS2R38* and variation in PTC sensitivity have been studied previously (Kim et al., 2003; Suzuki et al., 2010; Wooding et al., 2006). Three amino acid sites are responsible for PTC sensitivity in human TAS2R38 (hTAS2R38), at positions 49, 262, and 296. Two common variants are widely distributed worldwide: PAV (Proline 49, Alanine 262, and Valine 296), which encodes the PTC‐taster receptor, and AVI (Alanine 49, Valine 262, and Isoleucine 296), which encodes the PTC‐non‐taster receptor. The less common variants of hTAS2R38, namely AAI, PVI, and AAV, show intermediate sensitivity to PTC (Bufe et al., 2005). Few studies of nonhuman primates have explored the functional divergence of *TAS2R38* with respect to PTC sensitivity, including studies of *Macaca fuscata* and *Pan troglodytes* (Suzuki et al., 2010; Wooding et al., 2006).

Some studies have suggested that geographical separation enabled the independent divergence of TAS2R38 and bitter taste perception (Campbell et al., 2011; Hayakawa et al., 2012; Suzuki‐Hashido et al., 2015). For example, in humans, the frequencies of TAS2R38 haplotypes associated with intermediate bitter taste sensitivity are higher in Africa than in other regions (Campbell et al., 2011). In some species of primates, such as *M. fuscata* and *P. troglodytes*, a PTC‐non‐sensitive haplotype has been found in only one specific area (Hayakawa et al., 2012; Suzuki‐Hashido et al., 2015).

**Figure 1:**
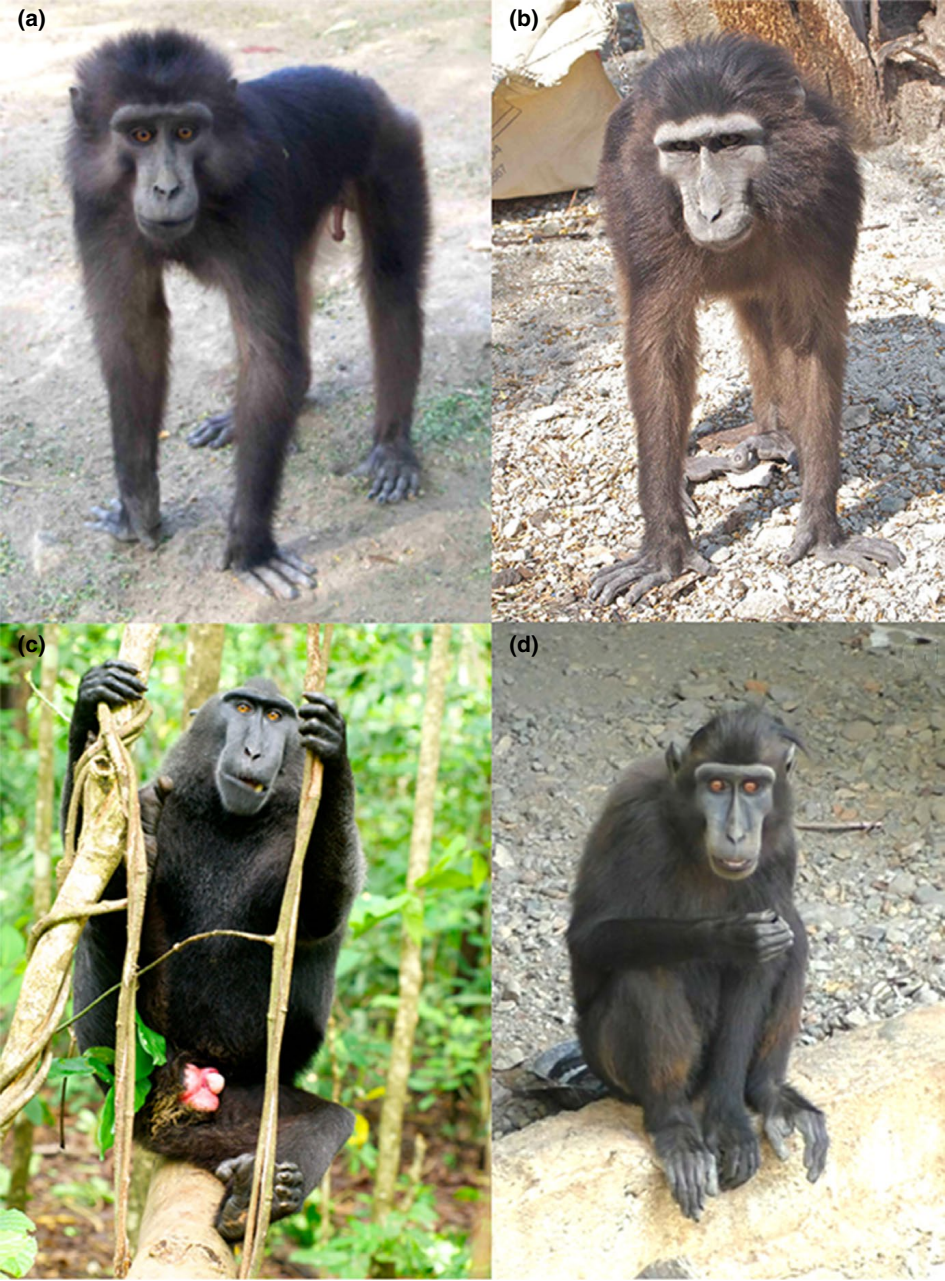
Photographs of Sulawesi macaques: *M. tonkeana* (a), *M. hecki* (b), *M. nigra* (c), and* M. nigrescens* (d)

Seven macaque species have an allopatric distribution on Sulawesi Island (Fooden, 1969). The Sulawesi macaques show considerable morphological variation (Figure 1) even though they inhabit a relatively small total area compared with other species of macaques (Fooden, 1969; Juliandi, Suryobroto, & Perwitasari‐Farajallah, 2009; Suryobroto, 1992). Although all Sulawesi macaques are frugivorous, there are some differences in the plant species they consume. For example, *M. tonkeana* occasionally consumes *Antidesma bunius*, while there are no reports of other Sulawesi macaques consuming this fruit. *M. nigra* also consumed *Dracontomelum dao*, unlike other Sulawesi macaques (O'Brien & Kinnaird, 1997; Riley, 2007).

A notable feature of Sulawesi Island is that it comprises three major tectonic subdivisions, two that originated in the Asian and Australian continental margins and one that emerged by orogeny due to the tectonic collision between two plates (Spakman & Hall, 2010). Accordingly, geological, geographical, and ecological conditions vary within the island, such as soil conditions and the composition of natural vegetation (Whitten & Henderson, 2012). Based on the geographical and ecological variations on Sulawesi Island and the slight differences in food preferences among Sulawesi macaques, we hypothesized that TAS2R38 also diverged among Sulawesi macaque species.

In this study, we performed a behavioral experiment, molecular genetic analysis, and in vitro functional analysis to characterize the divergence of TAS2R38 in four species of Sulawesi macaques. We found that TAS2R38 of Sulawesi macaques shows substantial individual variation in PTC taste perception both within and across species, and that this variation is caused by a variety of species‐specific genetic variants. Our analysis of interspecific and intraspecific variation in TAS2R38 in nonhuman primates improves our understanding of the evolution of bitter taste perception.

## 2 MATERIALS AND METHODS

### 2.1 Behavioral experiment

Phenylthiocarbamide sensitivity was analyzed using 54 captive individuals of four species of Sulawesi macaques (*M. tonkeana*, 12 individuals; *M. hecki*, 15 individuals; *M. nigrescens*, 12 individuals; and *M. nigra*, 15 individuals) in Indonesia.

Macaques were given slices of apple (control) or slices of apple that were soaked overnight in test solution (2 mM PTC in water; modified from Suzuki et al., 2010). A trial began when a monkey put an apple slice in its mouth. Whether they ate (accepted) or spat out (rejected) the apple was recorded. The acceptance rate (%) was calculated for PTC and control apples. Proportion tests were implemented in the stats package in R v. 3.3.1 (R Core Team, https://www.R-project.org/) to compare the control and PTC treatments. This experiment was approved by the Animal Ethics Commission of the Research and Community Service Institute, Ministry of Research, Technology and Higher Education, Bogor Agricultural University (Permission number no. 35‐2016 IPB).

### 2.2 Genotyping of *TAS2R38*

Genomic DNA of Sulawesi macaques was extracted from buccal swab of monkeys using the DNeasy Blood and Tissue Kit (QIAGEN GmbH). Genomic DNA of *M. nemestrina* and *M. fascicularis* was extracted from blood samples using the QIAamp DNA Investigator Kit. We checked the relatedness among subjects by analyzing the noncoding sequences for three loci in exome data (Terai, Y., Takuno, S., Widayati, K. A., Purba, L. H. P. S., Yan, X., Imai, H., & Suryobroto, B., in prep.). We calculated heterozygosity values using DnaSP version 6 (Rozas et al., 2017) on chromosomes 10 and 20 across a total of 7,024 bp. Expected and observed heterozygosity values were analyzed by Bartlett's test using R version 3.6.1.

Primers described in a previous study Mm‐TAS2R38‐F, Mm‐TAS2R38‐R, Mm‐TAS2R38_inner‐F, and Mm‐TAS2R38_inner‐R (Suzuki‐Hashido et al., 2015) were used to amplify and sequence the entire coding region of the *TAS2R38* gene from each DNA sample. Amplification of *TAS2R38* was performed using ExTaq DNA Polymerase (Takara Bio Inc.) by PCR using the following conditions: initial denaturation at 94°C for 10 min, 45 cycles of denaturation at 94°C for 10 s, annealing at 56°C for 30 s, and extension at 72°C for 1 min, followed by a final extension at 72°C for 10 min. PCR products were sequenced using BigDye Terminator v. 3.1 (Applied Biosystems), and the sequencing products were separated by capillary electrophoresis using a 3130 Genetic Analyzer (Applied Biosystems).

### 2.3 TAS2R38 population and sequence analyses

Multisite haplotypes were constructed from sequence data using DnaSP v. 5.1 (Librado & Rozas, 2009). The genealogical relationships among haplotypes were constructed, rooted with the TAS2R38 sequence of the hamadryas baboon (*Papio hamadryas*; Accession number AY724835.1), pig‐tailed macaque (*M. nemestrina*; JQ272210 and seven individual samples from Sumatera), and one individual of *M. fascicularis* using the median‐joining algorithm implemented in NETWORK v. 5 (Bandelt, Forster, & Röhl, 1999).

### 2.4 Functional assay of TAS2R38

Calcium imaging methods were used to evaluate the functions of TAS2R38 of Sulawesi macaques (Purba et al., 2017; Suzuki‐Hashido et al., 2015). TAS2R38 was tagged at the N terminus with the first 45 amino acids of rat somatostatin receptor type 3 for cell‐surface targeting and at the C terminus with the last eight amino acids of bovine rhodopsin as an epitope tag. The tagged TAS2R38s were inserted into the mammalian expression vector pEAK10 (Edge BioSystems, Inc.) and transiently transfected to HEK293T cells with Gα16‐gust44 using Lipofectamine 2000 (Life Technologies, Inc.).

The phenylthiocarbamide was dissolved in assay buffer (130 mM NaCl, 10 mM glucose, 5 mM KCl, 2 mM CaCl_2_, 1.2 mM MgCl_2_, and 10 mM HEPES; pH 7.4) to make 0.1, 0.3, 1, 3, 10, 30, and 100 µM for functional assay. For the functional assay, Calcium 4 (Molecular Devices, Inc.) was used as an intracellular Ca^2+^ indicator. Fluorescence was measured at 525 nm following excitation at 485 nm at 2‐s intervals for 120 s at 27°C using the FlexStation 3 Microplate Reader (Molecular Devices Japan, Inc.). Ligand solution was added at 20 s.

The calcium response amplitudes are expressed as Δ*F*/*F*, which is the ratio of the ligand‐dependent increase in fluorescence to the fluorescence before ligand addition. The response of cells transfected with the empty pEAK10 vector and Gα16gust44 was defined as the mock response (TAS2R‐independent response) and was subtracted from all responses. Δ*F*/*F* values were fitted by the Hill equation (*y* = (max−min)/(1 + (*x*/EC50))) using the drc package in R (Ritz, Baty, Streibig, & Gerhard, 2015). Three independent measurements were conducted for each vector.

### 2.5 Cell culture

HEK293T cells were provided by Dr. Matsunami (Duke University) via Dr. Misaka (University of Tokyo) for the functional analysis. Cells were cultivated in a 5% CO_2_ incubator at 37°C with Dulbecco's Modified Eagle's Medium (Sigma‐Aldrich Japan) supplemented with 10% fetal bovine serum.

## 3 RESULTS

### 3.1 Behavioral experiment

In a behavioral experiment, we evaluated the responses of monkeys to 2 mM PTC‐soaked apples. *Macaca hecki* ate control apple slices (90%–100%) and rejected PTC‐treated apples (0%–20%; Prop.Test, *p* < .05, Table 1), consistent with previous findings for *M. fuscata* by Suzuki et al. (2010). *Macaca hecki* could discriminate between PTC‐treated and control apple slices, indicating that they are PTC tasters. In the three other species, most individuals rejected the PTC‐treated apples (Prop.Test, *p* < .05). However, three individuals of *M. tonkeana*, two individuals of *M. nigra*, and one individual of *M. nigrescens* ate both PTC‐treated apples (80%–100%) and control apples (100%; Prop.Test, *p* > .05). Other individuals accepted some proportion of PTC‐soaked apples (30%–75%, Prop.Test, *p* < .05), indicating an intermediate response to PTC (Table 1). These results suggested that there is divergence in bitter taste perception among the four species.

**Table 1:** Monkey performance in behavioral tests

No	Subject (code)	Sex	Age	PTC Treated			Control			*p*‐value^a^	Amino acid haplotype (diploid)
				Acceptance	Trial	%	Acceptance	Trial	%		
1	*M. hecki (He01)*	Male	Young adult	0	10	0	12	12	100	9.27 × 10^−38^	GG
2	*M. hecki (He02)*	Female	Young adult	0	10	0	12	12	100	9.27 × 10^−38^	AA
3	*M. hecki (He03)*	Male	Juvenile	0	4	0	6	6	100	3.34 × 10^−13^	AI
4	*M. hecki (He04)*	Female	Juvenile	0	8	0	11	11	100	1.56 × 10^−29^	AD
5	*M. hecki (He05)*	Female	Juvenile	0	9	0	11	11	100	1.21 × 10^−33^	AD
6	*M. hecki (He06)*	Male	Adult	0	10	0	17	18	94.44	8.51 × 10^−38^	AI
7	*M. hecki (He07)*	Male	Adult	0	5	0	9	10	90	3.06 × 10^−17^	FF
8	*M. hecki (He08)*	Male	Juvenile	1	5	20	10	10	100	2.2 × 10^−10^	AA
9	*M. hecki (He09)*	Male	Adult	0	10	0	10	10	100	9.35 × 10^−38^	AI
10	*M. hecki (He10)*	Female	Adult	0	10	0	12	12	100	9.27 × 10^−38^	AD
11	*M. hecki (He11)*	Male	Juvenile	0	10	0	11	12	91.67	9.27 × 10^−38^	AA
12	*M. hecki (He12)*	Male	Juvenile	0	11	0	12	12	100	7.10 × 10^−42^	AI
13	*M. hecki (He13)*	Male	Juvenile	0	1	0	2	2	100	.051	AE
14	*M. hecki (He14)*	Female	Adult	0	15	0	15	15	100	2.29 × 10^−58^	AA
15	*M. hecki (He15)*	Female	Adult	0	15	0	19	19	100	2.14 × 10^−58^	AA
16	*M. nigra (Nig01)*	Male	Young adult	10	10	100	11	11	100	.99	TT
17	*M. nigra (Nig02)*	Male	Juvenile	0	10	0	16	16	100	8.81 × 10^−38^	AF
18	*M. nigra (Nig03)*	Female	Juvenile	0	8	0	12	13	92.31	1.53 × 10^−29^	BO
19	*M. nigra (Nig04)*	Female	Juvenile	0	11	0	12	12	100	7.10 × 10^−42^	TO
20	*M. nigra (Nig05)*	Male	Adult	0	10	0	11	11	100	9.32 × 10^−38^	AO
21	*M. nigra (Nig06)*	Female	Adult	0	15	0	14	14	100	2.33 × 10^−58^	AO
22	*M. nigra (Nig07)*	Male	Adult	0	3	0	2	2	100	1.66 × 10^−9^	OO
23	*M. nigra (Nig08)*	Female	Juvenile	0	10	0	10	10	100	9.35 × 10^−38^	AF
24	*M. nigra (Nig09)*	Female	Juvenile	0	10	0	11	11	100	9.32 × 10^−38^	KT
25	*M. nigra (Nig10)*	Male	Juvenile	0	11	0	11	11	100	7.15 × 10^−42^	HL
26	*M. nigra (Nig11)*	Female	Juvenile	0	10	0	10	10	100	9.35 × 10^−38^	AO
27	*M. nigra (Nig12)*	Female	Juvenile	7	17	41.17	15	15	100	7.25 × 10^−21^	TT
28	*M. nigra (Nig13)*	Male	Adult	0	11	0	9	9	100	7.15 × 10^−42^	AT
29	*M. nigra (Nig14)*	Male	Young adult	0	10	0	13	13	100	9.18 × 10^−38^	OO
30	*M. nigra (Nig15)*	Male	Juvenile	15	16	93.75	15	15	100	.90	TT
31	*M. nigrescens (Nigc01)*	Male	Juvenile	0	10	0	14	14	100	9.07 × 10^−38^	FF
32	*M. nigrescens (Nigc02)*	Female	Adult	0	11	0	12	12	100	7.10 × 10^−42^	AA
33	*M. nigrescens (Nigc03)*	Male	Adult	0	10	0	13	13	100	9.18 × 10^−38^	AA
34	*M. nigrescens (Nigc04)*	Female	Adult	0	10	0	10	10	100	9.35 × 10^−38^	CF
35	*M. nigrescens (Nigc05)*	Female	Juvenile	0	10	0	12	12	100	9.27 × 10^−38^	AO
36	*M. nigrescens (Nigc06)*	Male	Adult	0	8	0	9	9	100	1.56 × 10^−29^	OO
37	*M. nigrescens (Nigc07)*	Male	Juvenile	0	16	0	13	14	92.86	1.76 × 10^−62^	FF
38	*M. nigrescens (Nigc08)*	Female	Adult	0	11	0	19	19	100	6.41 × 10^−42^	FO
39	*M. nigrescens (Nigc09)*	Female	Juvenile	0	11	0	11	11	100	7.15 × 10^−42^	AF
40	*M. nigrescens (Nigc10)*	Male	Juvenile	0	15	0	20	20	100	2.10 × 10^−58^	AO
41	*M. nigrescens (Nigc11)*	Male	Juvenile	22	22	100	21	21	100	7.23 × 10^–1^	UU
42	*M. nigrescens (Nigc12)*	Female	Juvenile	0	10	0	12	13	92.31	9.18 × 10^−38^	FO
43	*M. tonkeana (To01)*	Male	Juvenile	10	10	100	10	10	100	1.00	QR
44	*M. tonkeana (To02)*	Female	Adult	0	3	0	5	5	100	3.37 × 10^−9^	IM
45	*M. tonkeana (To03)*	Female	Juvenile	9	27	33.33	29	29	100	4.97 × 10^−45^	IQ
46	*M. tonkeana (To04)*	Female	Juvenile	0	2	0	2	4	50	3.88 × 10^−7^	AJ
47	*M. tonkeana (To05)*	Male	Young adult	1	10	10	10	10	100	5.52 × 10^−30^	AR
48	*M. tonkeana (To06)*	Female	Adult	1	10	10	13	13	100	5.42 × 10^−30^	II
49	*M. tonkeana (To07)*	Male	Adult	10	10	100	10	10	100	1.00	SS
50	*M. tonkeana (To08)*	Male	Juvenile	0	12	0	9	10	90	5.47 × 10^−46^	NR
51	*M. tonkeana (To09)*	Male	Adult	21	28	75	27	27	100	4.28 × 10–5	NR
52	*M. tonkeana (To10)*	Male	Juvenile	0	10	0	12	12	100	9.27 × 10^−38^	RR
53	*M. tonkeana (To11)*	Female	Adult	0	10	0	11	11	100	9.32 × 10^−38^	NP
54	*M. tonkeana (To12)*	Male	Adult	13	16	81.25	16	16	100	.14	RR

Note

A trial is defined as the act of a monkey putting an apple slice into its mouth, followed by acceptance or rejection of the slice.

a Probability that the proportion of acceptance in the PTC‐treated session is the same as that in the control session.

### 3.2 Genotyping

**Figure 2:**
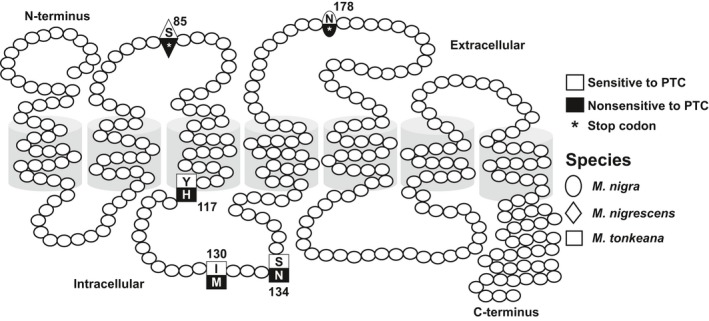
Schematic transmembrane topology of the TAS2R38 receptor of Sulawesi macaques. The structure is based on the structure of bovine rhodopsin. Black labels indicate the cause of PTC‐non/PTC‐low sensitivity in four species Sulawesi macaques. Different symbols indicate different species

We determined the nucleotide sequences of *TAS2R38* to investigate the genetic basis of PTC‐sensitive and PTC‐non‐sensitive behaviors. From 59 individuals (including additional nucleotide sequences of five individuals of *M. hecki*), we identified 29 alleles based on combinations of eight synonymous and 23 nonsynonymous mutations and three insertion and deletion mutations (Table 2), designated h_01–h_29. The alleles encoded 21 different TAS2R38 amino acid sequences (protein haplotypes), designated A–U. In *M. nigra*, the PTC‐non‐sensitive allele had a frameshift mutation caused by an insertion of a G nucleotide at site 492, which induced a premature stop codon at nucleotide position 534 (haplotype T). In *M. nigrescens*, the PTC‐non‐sensitive allele lost two nucleotides at positions 176 and 177, which induced a premature stop codon at nucleotide position 253 (haplotype U). In *M. tonkeana*, the PTC‐non‐sensitive alleles possessed nonsynonymous nucleotide mutations leading to specific amino acids at sites 117, 130, 134, and 315 (haplotypes P, Q, R, and S) compared with the wild‐type PTC‐sensitive alleles (Figure 2).

**Table 2:** Variable sites in TAS2R38 alleles of 59 Sulawesi macaques, eight pig‐tailed macaques, and one long‐tailed macaque

Amino acid haplotype	Nucleotide haplotype	Nucletide change	A6A	A7A,G	C10G	C25A	A49G	G67A	T108T	C129T	G144A	C59S	177	C178G	C189T	G191A	C232T	A239G	A166G	C270T	G280A	C281T	M100I	299	300	C305T	G331A	T349C	C390G	G401A	A412G	A420C	T432C
		Amino acid change	L2L	T3A	L4V	S12Y	T17A	V23I	I36I	D43D	Q48Q			L60V	S63S	R64Q	L78F	H80R	N89S	H90H	A94T	V94A				A102V	A111T	Y117H	I130M	N134S	R138G	I140I	L144L
		Species	6	7	10	35	49	67	108	129	144	176		178	189	191	232	239	266	270	280	281	298			305	331	349	390	401	412	420	432
A	Hap_01	*M. hecki*; *M. nigra*; *M. nigrescens*; *M. tonkeana*	A	A	C	C	A	G	T	C	G	G	T	C	C	G	C	A	A	C	G	C	A	T	G	C	G	T	C	A	A	A	C
	Hap_02	*M. hecki*; *M. nigra*; *M. nigrescens*	.	.	.	.	.	.	.	.	.	.	.	.	.	.	.	.	.	.	.	.	.	.	.	.	.	.	.	.	.	.	.
	Hap_03	*M. hecki*	.	.	.	.	.	.	.	.	.	.	.	.	.	.	.	.	.	.	.	.	.	.	.	.	.	.	.	.	.	.	.
	Hap_04	*M. hecki*	.	.	.	.	.	.	.	.	A	.	.	.	.	.	.	.	.	.	.	.	.	.	.	.	.	.	.	.	.	.	.
	Hap_05	*M. tonkeana*	.	.	.	.	.	.	.	.	.	.	.	.	.	.	.	.	.	.	.	.	.	.	.	.	.	.	.	.	.	.	.
B	Hap_06	*M. nigra*	.	.	.	.	G	.	.	.	.	.	.	.	.	A	.	.	.	.	.	.	*	*	*	.	.	.	.	.	.	.	.
C	Hap_07	*M. nigrescense*	.	.	.	.	.	.	.	.	.	.	.	.	.	.	T	.	.	.	.	.	.	.	.	T	.	.	.	.	.	.	.
D	Hap_08	*M. hecki*	.	.	.	.	.	.	.	.	.	.	.	.	.	.	.	G	.	.	.	.	.	.	.	.	.	.	.	.	.	.	.
E	Hap_09	*M. hecki*	.	.	.	.	.	.	.	.	.	.	.	.	.	.	.	.	G	.	.	.	.	.	.	.	.	.	.	.	.	.	.
	Hap_10	*M. hecki*	.	.	.	.	.	.	.	.	.	.	.	.	.	.	.	.	G	.	.	.	.	.	.	.	.	.	.	.	.	.	.
F	Hap_11	*M. hecki*;* M. nigra*; *M. nigrescens*	.	.	.	.	.	.	.	.	.	.	.	.	.	.	.	.	.	.	.	.	.	.	.	T	.	.	.	.	.	.	.
G	Hap_12	*M. hecki*	.	.	.	.	.	.	.	.	.	.	.	.	.	.	.	.	.	.	.	.	.	.	.	T	.	.	.	.	.	.	.
H	Hap_13	*M. nigra*	.	.	.	.	.	.	.	.	.	.	.	.	.	.	.	.	.	.	.	.	.	.	.	.	.	.	.	.	.	.	.
I	Hap_14	*M. hecki*; *M. tonkeana*	.	.	.	.	.	.	.	.	.	.	.	.	.	.	.	.	.	.	.	.	.	.	.	.	.	.	.	.	.	.	.
J	Hap_15	*M. tonkeana*	.	.	.	.	.	.	.	.	.	.	.	.	.	.	.	.	.	.	.	.	.	.	.	.	.	.	.	.	.	.	.
K	Hap_16	*M. nigra*	.	.	.	.	.	.	.	.	.	.	.	.	.	.	.	.	.	.	.	.	.	.	.	.	.	.	.	.	.	.	.
L	Hap_17	*M. nigra*	.	.	.	.	.	.	.	.	.	.	.	.	.	.	.	.	.	.	.	.	.	.	.	.	.	.	.	.	.	.	.
M	Hap_18	*M. tonkeana*	.	.	.	.	.	.	.	T	.	.	.	.	T	.	.	.	.	.	.	.	.	.	.	.	.	.	.	.	.	.	.
N	Hap_19	*M. tonkeana*	.	.	.	.	.	.	.	.	.	.	.	.	T	.	.	.	.	.	.	.	.	.	.	.	.	.	.	.	.	.	.
O	Hap_20	*M. hecki*; *M. nigra*; *M. nigrescens*	.	.	.	.	.	.	.	.	.	.	.	.	.	.	.	.	.	.	.	.	.	.	.	.	.	.	.	.	.	.	.
	Hap_21	*M. nigra*	.	.	.	.	.	.	.	.	.	.	.	.	.	.	.	.	.	.	.	.	.	.	.	.	.	.	.	.	.	.	.
P	Hap_22	*M. tonkeana*	.	.	.	.	.	.	.	.	.	.	.	.	.	.	.	.	.	.	A	.	.	.	.	.	.	C	G	G	.	.	.
Q	Hap_23	*M. tonkeana*; *M. nemestrina*	.	.	.	.	.	.	.	.	.	.	.	.	.	.	.	.	.	.	.	.	.	.	.	.	.	C	.	G	.	.	.
R	Hap_24	*M. tonkeana*	.	.	.	.	.	.	.	.	.	.	.	.	.	.	.	.	.	.	.	.	.	.	.	.	.	C	G	G	.	.	.
S	Hap_25	*M. tonkeana*	.	.	.	.	.	.	.	.	.	.	.	.	.	.	.	.	.	.	.	.	.	.	.	.	.	C	G	G	.	.	.
T	Hap_26	*M. nigra*	.	.	.	.	.	.	.	.	.	.	.	.	.	.	.	.	.	.	.	.	.	.	.	.	.	.	.	.	.	.	.
	Hap_27	*M. nigra*	.	.	.	.	.	.	.	.	.	.	.	.	.	.	.	.	.	.	.	.	.	.	.	.	.	.	.	.	.	.	.
	Hap_28	*M. nigra*	.	.	.	.	.	.	.	.	.	.	.	.	.	.	.	.	.	.	.	.	.	.	.	.	.	.	.	.	.	.	.
U	Hap_29	*M. nigrescense*	.	.	.	.	.	.	.	.	.	*	*	.	.	.	.	.	.	.	.	.	.	.	.	.	.	.	.	.	.	.	.
MnA	Hap_30	*M. nemestrina*	.	.	.	.	.	.	.	.	.	.	.	.	.	.	.	.	.	.	.	.	.	.	.	.	.	.	.	G	.	.	.
MnB	Hap_31	*M. nemestrina*	.	.	.	.	.	.	.	.	.	.	.	.	.	.	.	.	.	.	.	.	.	.	.	.	.	.	.	G	.	.	.
MnC	Hap_32	*M. nemestrina*	.	.	.	.	.	.	.	.	.	.	.	.	.	.	.	.	.	.	.	.	.	.	.	.	.	.	.	G	G	.	.
MnD	Hap_33	*M. nemestrina*	.	.	.	A	.	.	.	.	.	.	.	.	.	.	.	.	.	.	.	.	.	.	.	.	.	.	.	G	.	.	.
MnE	Hap_34	*M. nemestrina*	.	.	.	A	.	.	.	.	.	.	.	.	.	.	.	.	.	.	.	.	.	.	.	.	.	.	.	G	.	.	.
MnF	Hap_35	*M. nemestrina*	.	.	.	A	.	.	.	.	.	.	.	G	.	.	.	.	.	.	.	.	.	.	.	.	.	.	.	G	.	.	.
MfasA	Hap_36	*M. fascicularis*	.	.	.	A	.	.	.	.	.	.	.	.	.	.	.	.	.	.	.	.	.	.	.	.	.	.	.	G	.	.	.
MfasB	Hap_37	*M. fascicularis*	.	.	.	A	.	.	.	.	.	.	.	.	.	.	.	.	.	.	.	.	.	.	.	.	.	.	.	G	.	.	.
Paha	Hap_38	*Papio hamadryas*	G	.	.	A	.	.	A	.	.	.	.	.	.	.	.	.	.	.	.	T	.	.	.	.	.	.	.	G	.	C	T

Amino acid haplotype	Nucleotide haplotype	Nucletide change	C483T	G164G	506	507	T517G	519	520	A569C	G608A	G698A	T717C	A748G	C771T	G772A,T	C806T	T810G	G884A	C885T	G887A	C907T	G910C	A926C	C935G	T936C	G944A	G949A	G968A	C973T	C993T	T996C	G997A
		Amino acid change	C161C				T172T			N190H	V203I	D233N	L239P	I250V	A257V	I258I	L269L	L270L	A295T,V	A295T,V	V296I	K303N	L304L	T309T	L295P;V	L295P;V	A315T	S317S	A323T	H325H	T331I	L332P	C333C
		Species	483	492			516			568	607	697	716	748	770	771	805	810	883	884	886	906	909	925	934	935	943	948	967	972	992	995	996
A	Hap_01	*M. hecki*; *M. nigra*; *M. nigrescens*; *M. tonkeana*	C	–	A	C	T	G	C	A	G	G	T	A	C	G	C	T	G	C	G	C	G	A	C	T	G	G	G	C	C	T	G
	Hap_02	*M. hecki*; *M. nigra*; *M. nigrescens*	.	–	.	.	.	.	.	.	.	.	.	.	.	A	T	.	.	.	.	.	.	.	.	.	.	.	.	.	.	.	.
	Hap_03	*M. hecki*	.	–	.	.	.	.	.	.	.	.	.	.	.	.	.	.	.	.	.	T	.	.	.	.	.	.	.	.	.	.	.
	Hap_04	*M. hecki*	.	–	.	.	.	.	.	.	.	.	.	.	.	.	.	.	.	.	.	T	.	.	.	.	.	.	.	.	.	.	.
	Hap_05	*M. tonkeana*	.	–	.	.	.	.	.	.	.	.	.	.	.	T	.	.	.	.	.	.	.	.	.	.	.	.	.	.	.	.	.
B	Hap_06	*M. nigra*	.	–	.	.	.	.	.	.	.	.	.	.	.	.	.	.	.	.	.	.	.	.	.	.	.	.	.	.	.	.	.
C	Hap_07	*M. nigrescense*	.	–	.	.	.	.	.	.	.	.	.	.	.	.	.	.	.	.	.	.	.	.	.	.	.	.	.	.	.	C	.
D	Hap_08	*M. hecki*	.	–	.	.	.	.	.	.	.	.	.	.	.	A	T	.	.	.	.	.	.	.	.	.	.	.	.	.	.	.	.
E	Hap_09	*M. hecki*	T	–	.	.	.	.	.	.	.	.	.	.	.	.	.	.	.	.	.	.	.	.	.	.	.	.	.	.	.	.	.
	Hap_10	*M. hecki*	.	–	.	.	.	.	.	.	.	.	.	.	.	.	.	.	.	.	.	.	.	.	.	.	.	.	.	.	.	.	.
F	Hap_11	*M. hecki*; *M. nigra*; *M. nigrescens*	.	–	.	.	.	.	.	.	.	.	.	.	.	.	.	.	.	.	.	.	.	.	.	.	.	.	.	.	.	C	.
G	Hap_12	*M. hecki*	.	–	.	.	.	.	.	.	.	.	.	.	.	.	.	.	.	.	.	.	.	.	.	.	.	.	.	.	.	.	.
H	Hap_13	*M. nigra*	.	–	.	.	.	.	.	.	.	A	C	.	T	.	.	.	A	.	A	.	.	.	.	.	.	A	A	.	.	.	.
I	Hap_14	*M. hecki*; *M. tonkeana*	.	–	.	.	.	.	.	.	.	.	.	.	T	.	.	.	.	.	A	.	.	.	.	.	.	.	.	.	.	.	.
J	Hap_15	*M. tonkeana*	.	–	.	.	.	.	.	.	.	.	.	.	T	.	.	.	.	.	.	.	.	.	.	C	.	.	.	.	.	.	.
K	Hap_16	*M. nigra*	.	–	.	.	.	.	.	.	.	.	.	.	.	.	.	.	.	.	.	.	.	C	G	.	.	.	.	.	.	.	.
L	Hap_17	*M. nigra*	.	–	.	.	.	.	.	.	.	.	.	.	.	.	.	.	A	.	A	.	.	.	.	.	.	A	.	.	.	.	.
M	Hap_18	*M. tonkeana*	.	–	.	.	.	.	.	.	.	.	.	.	.	.	.	.	.	T	.	.	.	.	.	.	A	.	.	.	.	.	.
N	Hap_19	*M. tonkeana*	.	–	.	.	.	.	.	.	.	.	.	.	.	.	.	.	.	.	.	.	.	.	.	.	A	.	.	.	.	.	.
O	Hap_20	*M. hecki*; *M. nigra*; *M. nigrescens*	.	–	.	.	.	.	.	.	.	.	.	.	.	.	.	.	.	.	.	.	.	.	.	.	.	.	.	.	.	C	.
	Hap_21	*M. nigra*	.	–	.	.	.	.	.	.	.	.	.	.	.	.	.	.	.	.	.	.	.	.	.	.	.	.	.	.	.	C	A
P	Hap_22	*M. tonkeana*	.	–	.	.	.	.	.	.	.	.	.	.	.	.	.	.	.	.	.	.	.	.	.	.	.	.	.	.	.	.	.
Q	Hap_23	*M. tonkeana*; *M. nemestrina*	.	–	.	.	.	.	.	.	.	.	.	.	.	.	.	.	.	.	.	.	.	.	.	.	.	.	.	.	.	.	.
R	Hap_24	*M. tonkeana*	.	–	.	.	.	.	.	.	.	.	.	.	.	.	.	.	.	.	.	.	.	.	.	.	.	.	.	.	.	.	.
S	Hap_25	*M. tonkeana*	.	–	.	.	.	.	.	.	.	.	.	.	.	.	.	.	.	.	.	.	C	.	.	.	.	.	.	.	.	.	.
T	Hap_26	*M. nigra*	.	G	.	.	.	.	.	.	.	.	.	.	.	.	.	.	.	.	.	.	.	.	.	.	.	.	.	.	.	C	.
	Hap_27	*M. nigra*	.	G	.	.	.	.	.	.	.	.	.	.	.	.	.	.	.	.	.	.	.	.	G	.	.	.	.	.	.	.	.
	Hap_28	*M. nigra*	.	G	.	.	.	.	.	.	.	.	.	.	.	.	.	.	.	.	.	.	.	.	.	.	.	.	.	.	.	.	.
U	Hap_29	*M. nigrescense*	.	–	.	.	.	.	.	.	.	.	.	.	.	.	.	.	.	.	.	.	.	.	.	.	.	.	.	.	.	.	.
MnA	Hap_30	*M. nemestrina*	.	–	.	.	.	.	.	.	.	.	.	.	.	.	.	.	.	.	.	.	.	.	.	.	.	.	.	.	.	.	.
MnB	Hap_31	*M. nemestrina*	.	–	.	.	G	.	.	.	.	.	.	.	.	.	.	.	.	.	.	.	.	.	.	.	.	.	.	.	.	.	.
MnC	Hap_32	*M. nemestrina*	.	–	*	*	.	.	.	.	.	.	.	.	.	.	.	.	.	.	.	.	.	.	.	.	.	.	.	.	.	.	.
MnD	Hap_33	*M. nemestrina*	.	–	.	.	.	*	*	.	.	.	.	.	.	.	.	.	.	.	.	.	.	.	.	.	.	.	.	.	.	.	.
MnE	Hap_34	*M. nemestrina*	.	–	.	.	.	.	.	.	.	.	.	.	.	.	.	.	.	.	.	.	.	.	.	.	.	.	.	.	.	.	.
MnF	Hap_35	*M. nemestrina*	.	–	.	.	.	.	.	.	.	.	.	.	.	.	.	.	.	.	.	.	.	.	.	.	.	.	.	.	.	.	.
MfasA	Hap_36	*M. fascicularis*	.	–	.	.	.	.	.	.	.	.	.	.	T	.	.	.	.	.	.	.	.	.	.	.	.	.	.	.	.	.	.
MfasB	Hap_37	*M. fascicularis*	.	–	.	.	.	.	.	C	A	.	.	.	T	.	.	.	.	.	.	.	.	.	.	.	.	.	.	.	T	.	.
Paha	Hap_38	*Papio hamadryas*	.	–	.	.	.	.	.	.	.	.	.	G	T	.	.	G	.	.	.	.	.	.	.	.	.	.	.	.	.	.	.

Note

*, deletion; –, insertion.

**Figure 3:**
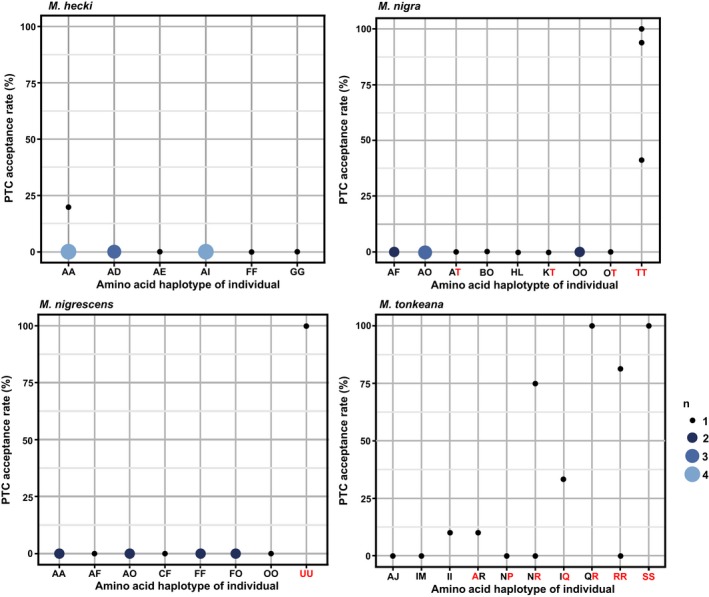
PTC acceptance rate for each amino acid haplotype of individuals of the four species of Sulawesi macaques. The *y*‐axis shows the PTC acceptance rate (%); the *x*‐axis shows the amino acid haplotype. Red indicates the PTC‐non‐taster/PTC‐low‐sensitivity haplotype

Individuals that were homozygous for nonsensitive alleles showed high PTC acceptance rates, while individuals with sensitive alleles showed very low PTC acceptance rates. However, one individual of *M. nigra* with homozygous nonsensitivity alleles rejected PTC and some homozygous and heterozygous individuals of *M. tonkeana* showed variation and intermediate responses to PTC (Figure 3). These findings were similar to observations of human TAS2R38 genotypes and behavioral responses and prompted us to examine the protein function (Bufe et al., 2005). We analyzed noncoding sequences from exome data to evaluate the relatedness among the subject animals. We found no difference between the expected and observed heterozygosity values in both *M. tonkeana* (*p* = .36) and *M. hecki* (*p* = .06; Bartlett's test). Thus, we conclude that there was no inbreeding among the subjects used in this experiment.

### 3.3 Functional analysis

**Figure 4:**
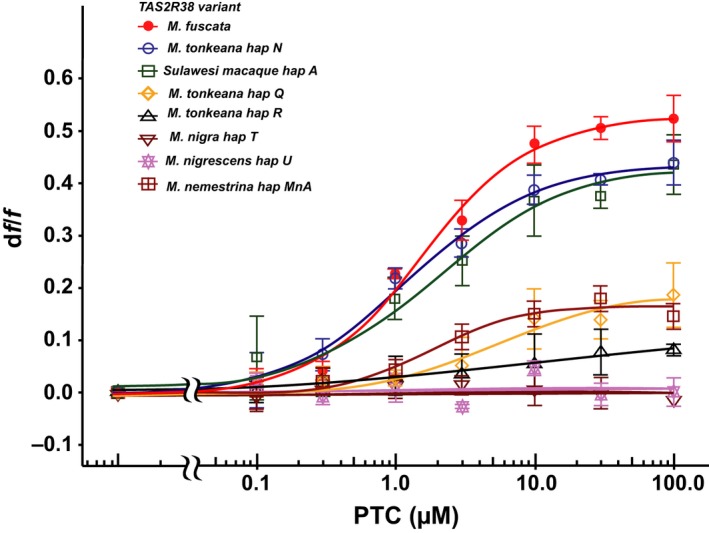
Dose–response curve for TAS2R38s of Sulawesi macaques, *M. nemestrina*, and *M. fuscata* against PTC concentrations. Each point represents the mean ± standard error of the mean (*SEM*) determined from three independent measurements

We evaluated receptor activity by in vitro functional assays of several TAS2R38 alleles of Sulawesi macaques. We tested the high‐frequency allele (haplotype A) and another variant that differed from haplotype A at site 315, one of the sites that was predicted to be responsible for receptor sensitivity (haplotype N). The TAS2R38s showed a significant dose‐dependent response to PTC, and the EC_50_ values (EC_50_ = 2.10 ± 0.46 for haplotype A and 1.22 ± 0.37 for haplotype N) were consistent with those for TAS2R38 of *M. fuscata* (EC_50_ = 1.58 ± 0.17; ANOVA, *p* > .05; Figure 4). In contrast, the translated TAS2R38 with a G insertion in *M. nigra* (haplotype T) and translated TAS2R38 with a 2‐bp deletion in *M. nigrescens* (haplotype U) failed to respond to PTC. TAS2R38 of *M. tonkeana* with specific amino acid substitutions at positions 117 and 134 (haplotype Q) and another TAS2R38 of *M. tonkeana* with specific amino acids at 117, 130, and 134 (haplotype R) showed weak responses to PTC (Figure 4). Although the EC_50_ values for the responses could not be calculated owing to the nonspecific response at more than 300 µM, the results of the functional assay were consistent with the analyses of PTC‐non‐sensitive individuals and nucleotide mutations.

## 4 DISCUSSION

### 4.1 Functional divergence of TAS2R38 in Sulawesi macaques

We observed functional divergence of Sulawesi macaque TAS2R38 with respect to responses to PTC. Most Sulawesi macaques are PTC‐sensitive, similar to other macaque species (Chiarelli, 1963; Suzuki‐Hashido et al., 2015). We also observed PTC‐non‐sensitive phenotypes which caused by truncated protein in some individuals in two species of Sulawesi macaques, *M. nigra* and *M. nigrescens*. Protein truncations resulting in a lack of sensitivity also exists in other primate species, such as chimpanzees, Japanese macaques, and some species of New World monkey (Suzuki et al., 2010; Wooding, 2012; Wooding et al., 2006). These results confirm the high divergence of *TAS2R38*.

In addition to the nonsensitive alleles of *M. nigra* and *M. nigrescens*, which encode truncated TAS2R38 receptors, we found *TAS2R38* alleles in *M. tonkeana* that express apparently intact TAS2R38 receptors but exhibit very weak responses compared with those of wild‐type Sulawesi TAS2R38. Similar intact receptors with low PTC sensitivity have been found in humans (Bufe et al., 2005). The amino acids responsible for low sensitivity in *M. tonkeana* were located in transmembrane domain 3 (TM3; amino acid 117) and the internal loop domain (130 and 134) of TAS2R38. In humans, some amino acids located in TM3 are involved in the PTC binding and receptor activation, while the internal loop mediates intracellular interactions with G proteins (Biarnés et al., 2010). Based on the change in charge from polar‐no charge (tyrosine) to polar‐positive charge (histidine) at site 117, we predict that site 117 is important for determining the sensitivity to PTC. Using MutationTaster and PolyPhen, we confirmed that the variant is potentially damaging with respect to protein function (Adzhubei et al., 2010; Schwarz, Cooper, Schuelke, & Seelow, 2014).

Moreover, we observed intermediate responses to PTC in homozygous and heterozygous individuals of *M. tonkeana*, consistent with human taster/nontaster heterozygotes (PAV/AVI genotype), which show intermediate sensitivity between those of taster and nontaster groups (Campbell et al., 2011). This may result from different levels of allelic expression (Bufe et al., 2005) and may also explain the behavioral variation in *M. tonkeana*. The different levels of expression of TAS2R38 may result in the ability to detect more ligands, although further studies are needed to evaluate this possibility. In humans, associations between the *TAS2R38* nontaster allele and bitter sensitivity to the tropical berry *Antidesma bunius* are the inverse of those to PTC, suggesting that the nontaster allele enables individual to taste compounds in the fruit (Risso, Sainz, Morini, Tofanelli, & Drayna, 2018). Thus, we predicted that alleles associated with low sensitivity to PTC in *M. tonkeana* could detect another ligand. Interestingly, *M. tonkeana* occasionally consumes *Antidesma bunius* (Riley, 2007). These feeding behaviors of *M. tonkeana* are related to the TAS2R38 genotype and geographical distributions of the macaque and plant species.

### 4.2 Haplotype network and TAS2R38 divergence in Sulawesi macaques

**Figure 5:**
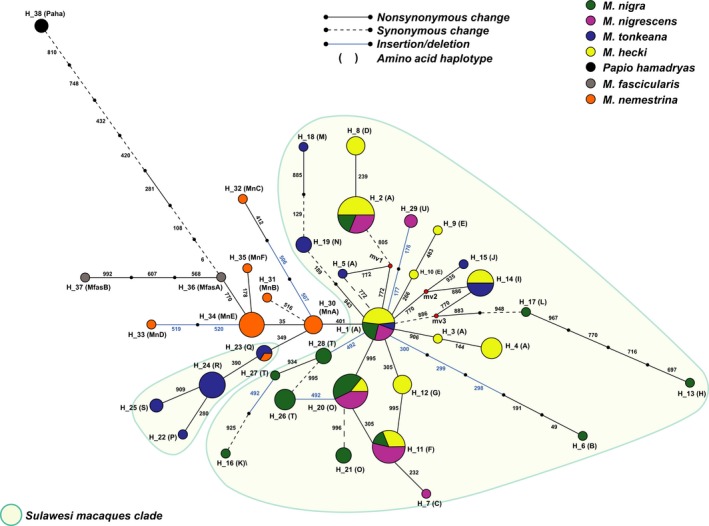
Median‐joining network for TAS2R38 alleles of Sulawesi macaques, pig‐tailed macaques, and long‐tailed macaques. Each circle represents a different haplotype and is shown along with the allele name. The size of a circle is proportional to the number of chromosomes. Nucleotide positions of mutations that differentiate alleles are indicated on the branches. Line styles indicate the mutation type. Font inside parenthesis represents amino acid haplotype

A haplotype network was constructed from 136 alleles (Figure 5). Hap_01 [hap_A] was shared between four species of Sulawesi macaques. This may be the ancestral haplotype of Sulawesi macaques. We found one haplotype that was shared between *M. tonkeana* and *M. nemestrina* (h_23 [haplotype_Q]). A haplotype of *M. nemestrina* (h_30 [hap_MnA]) was also closely related to two clades of Sulawesi macaques (h_1 [hap_A] and h_23 [hap_O]). These results imply that h_30 [hap MnA], h_01 [hap_A], and h_23 [hap_Q] are most closely related to the ancestral haplotype. It is not clear whether the ancestral haplotype still exists or has been lost. These results were consistent with those of previous studies indicating that the ancestor of Sulawesi macaques is closely related to *M. nemestrina* (Evans, Morales, Supriatna, & Melnick, 1999; Takenaka, Hotta, Kawamoto, Suryobroto, & Brotoisworo, 1987). We measured the receptor activity of the haplotype (h_30 [haplotype MnA]) by in vitro functional assays and found that it responded to PTC (EC_50_ = 1.88 ± 0.62; Figure 4).

We also observed three shared haplotypes in *M. hecki*, *M. nigra*, and *M. nigrescens* (hap_02 [hap A], hap_11[hap_F], and hap_20 [hap_O]). There was only one shared haplotype between *M. tonkeana* and *M. hecki* (hap_14 [hap_I]). Shared haplotypes may reflect geographical proximity and may predate the speciation event. The network also contained many haplotypes specific to individual Sulawesi macaque species. These results imply the adaptation of TAS2R38 to the environment, including recent adaptation to plants for feeding, soil types, and microbiomes related to the receptor (Whitten & Henderson, 2012).

Sulawesi macaques, which inhabit an island, possess high haplotype diversity, similar to other primates (Campbell et al., 2011; Hayakawa et al., 2012; Suzuki‐Hashido et al., 2015). The frequency of each haplotype was also different between species (Table 3). We observed seven amino acid haplotypes in *M. hecki*, eight in *M. nigra*, five in *M. nigrescens*, and nine in *M. tonkeana*. We analyzed the sequences for signatures of selection, but we did not obtain significance (Table 4). It is difficult to show selection using the number of individuals included in this study. In the near future, we will re‐evaluate selection using DNA from more individuals.

**Table 3:** Frequency haplotype for each Sulawesi macaque species

No	Amino acid haplotype	Nucleotide haplotype	*M. nigra*	*M. nigrescens*	*M. hecki*	*M. tonkeana*	*M. nemestrina*
1	A	Hap_01	0.10	0.125	0.15	0.042	0
2		Hap_02	0.10	0.208	0.2	0	0
3		Hap_03	0	0	0.025	0	0
4		Hap_04	0	0	0.125	0	0
5		Hap_05	0	0	0	0.042	0
6	B	Hap_06	0.033	0	0	0	0
7	C	Hap_07	0	0.042	0	0	0
8	D	Hap_08	0	0	0.1	0	0
9	E	Hap_09	0	0	0.025	0	0
10		Hap_10	0	0	0.025	0	0
11	F	Hap_11	0.067	0.292	0.1	0	0
12	G	Hap_12	0	0	0.1	0	0
13	H	Hap_13	0.033	0	0	0	0
14	I	Hap_14	0	0	0.1	0.167	0
15	J	Hap_15	0	0	0	0.042	0
16	K	Hap_16	0.033	0	0	0	0
17	L	Hap_17	0.033	0	0	0	0
18	M	Hap_18	0	0	0	0.042	0
19	N	Hap_19	0	0	0	0.125	0
20	O	Hap_20	0.20	0.250	0.050	0	0
21		Hap_21	0.10	0	0	0	0
22	P	Hap_22	0	0	0	0.042	0
23	Q	Hap_23	0	0	0	0.083	0.0625
24	R	Hap_24	0	0	0	0.333	0
25	S	Hap_25	0	0	0	0.083	0
26	T	Hap_26	0.167	0	0	0	0
27		Hap_27	0.033	0	0	0	0
28		Hap_28	0.10	0	0	0	0
29	U	Hap_29	0	0.083	0	0	0
30	MnA	Hap_30	0	0	0	0	0.25
31	MnB	Hap_31	0	0	0	0	0.063
32	MnC	Hap_32	0	0	0	0	0.063
33	MnD	Hap_33	0	0	0	0	0.063
34	MnE	Hap_34	0	0	0	0	0.438
35	MnF	Hap_35	0	0	0	0	0.063

**Table 4:** Tajima's *D* and Fu and Li's *D* tests of Sulawesi macaque TAS2R38

Species	*N*	*N* chromosome	Number of variable sites (S)	Number of haplotypes, h	Nucleotide diversity, Pi	Tajima's *D*	*p*‐value Tajima	Fu and Li's D*test statistic	*p*‐value Fu and Li's D*test statistic	Fu and Li's F*test statistic	*p*‐value Fu and Li's F*test statistic
*M. hecki*	20	40	11	11	0.00263	0.06146	>.10	0.89754	>.10	0.73911	>.10
*M. tonkeana*	12	24	13	10	0.00334	−0.13657	>.10	−0.5602	>.10	−0.50497	>.10
*M. nigrescens*	12	24	5	7	0.00174	0.87398	>.10	0.33154	>.10	0.56433	>.10
*M. nigra*	15	30	16	12	0.00219	−1.55972	>.10	−1.05583	>.10	−1.42581	>.10
*M. nemestrina*	8	16	4	7	0.0009	−0.79276	>.10	−1.52158	>.10	−1.51891	>.10

**Figure 6:**
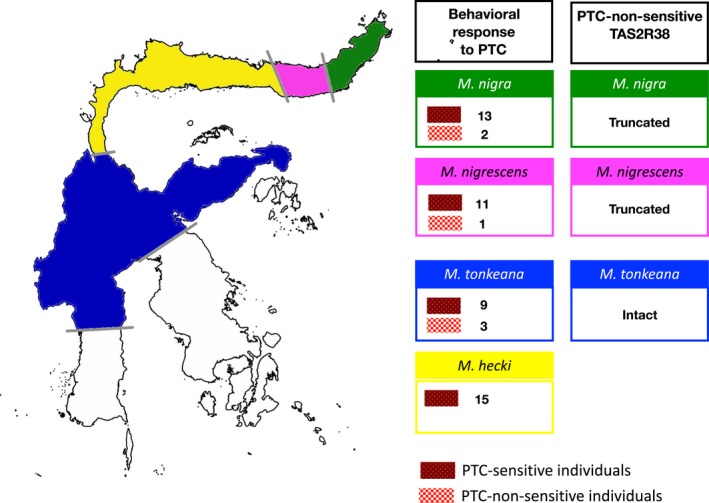
Overview of TAS2R38 diversity in Sulawesi macaques

This variation of TAS2R38 may be related to differences in the role of the receptor in dietary and nondietary biological processes. There are interspecific differences in diet (O'Brien & Kinnaird, 1997; Riley, 2007). TAS2R38 variation results in a range of responses to dietary sources of bitter compounds and facilitates adaptation to limited food resources in each habitat. With respect to nondietary biological processes, previous studies of humans have shown that TAS2R38 is involved in innate immunity (Lee et al., 2012). Thus, TAS2R38 variation may also have a role in the immune response of Sulawesi macaques. We predict that fragmentation played an important role in determining the distribution of genetic variation in TAS2R38 in Sulawesi macaques (Figure 6).

## CONFLICT OF INTEREST

None declared.

## AUTHOR CONTRIBUTIONS

K.A.W. conducted experiments, wrote the original draft, and analyzed and interpreted the data. Y.X. conducted experiments and analyzed and interpreted the data. N.S.‐H., A.I., L.H.P.S.P., and F.B. conducted experiments and analyzed the data. B.S. and Y.T. designed the experiments, wrote the paper, and revised the draft. H.I. designed the experiments and wrote the paper and finalized the manuscript. All authors agree to be held accountable for the content in the manuscript and approve the final version.

## Data Availability

DNA sequences: DDBJ accessions with entry ID LC473429 to LC473475. Original functional data available from the Dryad Digital Repository: https://doi.org/10.5061/dryad.908jf3r
